# Healthcare Workers’ Views on Approaches to Discussing Alternative Treatment Options in Multidisciplinary Cancer Care

**DOI:** 10.7759/cureus.102554

**Published:** 2026-01-29

**Authors:** Marco A Santos Teles, George Gayed, Vadim M Shteyler, Richard J Contrada, Dirk Moore, Francis Barchi, Paul R Duberstein, Malcolm D Mattes

**Affiliations:** 1 Department of Radiation Oncology, Rutgers University New Jersey Medical School, Newark, USA; 2 Department of Neurology, Montefiore Medical Center, Wakefield Campus, New York, USA; 3 Division of Pulmonary, Critical Care, Allergy, and Sleep Medicine, University of California San Francisco, San Francisco, USA; 4 Department of Health Behavior, Society, and Policy, Rutgers University, Piscataway, USA; 5 Department of Biostatistics and Epidemiology, Rutgers University, New Brunswick, USA; 6 Edward J. Bloustein School of Planning and Public Policy, Rutgers University, New Brunswick, USA; 7 Department of Psychology, Rutgers University, Piscataway, USA; 8 Department of Radiation Oncology, Rutgers Cancer Institute of New Jersey, New Brunswick, USA

**Keywords:** clinical oncology, decision making, informed consent, multidisciplinary discussion, su (1): shared-decision making

## Abstract

Purpose

Information about curative treatment options with comparable efficacy (e.g. surgery or radiation) is often not fully provided to patients when those options fall outside the scope of practice of the oncologist(s) who directly see a patient. This study evaluates healthcare workers’ views on the acceptability of this approach compared to others.

Methods

An electronic survey was sent to all physicians, trainees, nurses, therapists, researchers, and administrators at a cancer center. Respondents were asked to imagine being diagnosed with a potentially life-threatening illness that can be treated and cured in two standard ways, with similar efficacy but different side-effect profiles and impact on short- and long-term quality-of-life (Procedure A performed by Doctor A; Procedure B performed by Doctor B). Likert-type scales ranging from one (completely unacceptable) to five (completely acceptable) assessed Doctor A’s approach to discussing treatment options in each scenario.

Results

In this study, 266 individuals responded to the questionnaire (response rate 16%). The mean (standard deviation) Likert-type score was 1.58 (±0.93) for Approach #1 where Doctor A only presents Procedure A; 1.75 (±0.98) for Approach #2 where Doctor A presents both options but favors Procedure A; 2.54 (±1.35) for Approach #3 where Doctor A presents both options but favors Procedure A despite offering a referral to Doctor B to discuss Procedure B; and 4.75 (±0.76) for Approach #4 where Doctor A routinely refers all patients to Doctor B so each treatment option is discussed with the physician who performs each. Each of these differences were statistically significant (*p*<0.01).

Conclusion

A balanced, multidisciplinary approach to discussing treatment options is strongly favored by a variety of sampled healthcare workers. This is consistent with improving quality of healthcare delivery and patients' experience.

## Introduction

Guidelines for improving healthcare delivery and the patient care experience require physicians to inform patients of all available treatment options with comparable efficacy. For example, the need for transparency and providing sufficient information to facilitate informed decision-making by patients is one of the 10 new rules proposed by the Institute of Medicine’s Quality of Health Care Committee to guide patient-clinician relationships [1]. Furthermore, the Institute for Healthcare Improvement’s Triple Aim - improving the care experience, improving population health, and reducing costs of care - highlights the importance of informing patients of the benefits and limitations of healthcare practices through transparency and shared decision-making [2].

An effective informed consent process that includes a patient’s understanding of the possible risks, benefits, and reasonable alternatives to a given treatment is a critical component of achieving these goals [3]. Unfortunately, informed consent is often not well implemented by physicians [1,4]. For valid informed consent, physicians have an ethical obligation and common law duty to honestly disclose all significant medical information they possess, or reasonably should possess, that a patient would find material to making an informed decision, in the absence of controlling influences such as coercion or manipulation [4]. This may be particularly challenging in situations where alternative treatment options for a given medical condition fall outside of the scope of training and practice of a clinician [5], and the patient is dependent upon that clinician for appropriate multidisciplinary referrals to ensure that they learn about all of their treatment options from those trained in their use. This barrier to informed consent arises in multiple areas of medicine and is particularly common in oncology [5-7].

Oncology education in the United States is compartmentalized, with medical, surgical, and radiation oncology training conducted in 'siloed' programs specific to their treatment modality, and with no requirements from the Accreditation Council for Graduate Medical Education (ACGME) for cross-subspecialty training throughout graduate medical education [8,9]. As a result, though each type of oncologist is expected to have a high level of understanding of their own specific treatment modality (i.e. systemic therapy, surgery, or radiation therapy), it cannot necessarily be assumed that each can provide a thorough assessment of the risks and benefits of other treatment modalities in which they are not formally trained or board certified to use [10]. Although all physicians should practice evidence-based and guidelines-adherent medicine, the interpretation of evidence and guidelines is also inevitably subjective. As such, in situations where different treatment modalities can reasonably be offered to a patient, there is high potential for any individual oncologist to present an incomplete assessment of all available treatment options to a patient in favor of their own treatment modality.

Unfortunately, there is a significant amount of evidence demonstrating that these types of biases commonly dictate referral patterns, and subsequent patient care [11-18]. This has been documented in the context of decision-making between comparably effective local therapies (e.g. surgery or radiation) for curative treatment of non-metastatic solid tumors like prostate, bladder, lung, liver, and head/neck cancers [11-18] between the use of definitive radiation versus systemic therapy for certain lymphomas [19-20], and between the use of a potentially curative versus palliative approach for locally advanced but non-metastatic solid tumors [21,22]. Some examples from population-based studies of United States Medicare beneficiaries highlight the magnitude of the problem. For instance, only 44% of men with clinically localized prostate cancer are referred to a radiation oncologist to consider definitive radiation therapy as a standard-of-care treatment option [12]; the odds of a patient with stage III non-small cell lung cancer receiving National Comprehensive Cancer Network (NCCN) guideline-based therapies is three to 10 times higher if a patient has seen a radiation oncologist in addition to a medical oncologist [21]; and only 33% of patients with low-grade, early-stage lymphomas are treated with radiation therapy despite the therapy being known to substantially improve disease-specific and overall survival [19,20]. While multidisciplinary tumor board conferences promote informed physicians through the sharing of perspectives from multiple oncology subspecialties, these conferences do not necessarily ensure informed patients. This is because there is no certainty that the oncologist who initially saw a patient and presented their case in the tumor board will choose to follow the advice of their colleagues, discuss that advice with the patient, or refer the patient for a consultation so they can hear multiple opinions firsthand [5,7]. All together, these factors may have a significant impact on the completeness of the information made available to a patient to consider in decision-making. When treatment options are comparable, a patient’s values and preferences determine decisions more so than science; valid informed consent ensures that those values and preferences are the patient’s and not the clinician’s [19].

The purpose of this study was to determine healthcare workers’ views of different, commonly used approaches to discussing alternative treatment options with patients as part of an informed consent process, as an initial step toward developing more effective ways to promote multidisciplinary patient-centered cancer care. We further sought to evaluate how healthcare workers' views change when treatment options differ in efficacy and toxicity.

Components of this article were published as an abstract at the 2022 American Society for Radiation Oncology (ASTRO) Annual Meeting held in San Antonio, TX, on October 23-26, 2022, and the 2023 American Society of Clinical Oncology (ASCO) Annual Meeting, held in Chicago, IL, on June 2-6, 2023.

## Materials and methods

Survey design

Research Electronic Data Capture (REDCap; powered by Vanderbilt University (https://project-redcap.org/; REDCap application is free for non-profit, academic, and governmental institutions who join its consortium) was used to build an electronic survey, which is provided in the Appendices. The first section of the survey sought to assess what respondents viewed as the proper approach to discussing treatment options with patients. Respondents were asked to imagine being diagnosed with a potentially life-threatening medical disease that can be treated by two standard procedures: Procedure A performed by Doctor A or Procedure B performed by Doctor B, each with similar efficacy but different short-term and long-term side effects, and different impacts on the patient's quality of life. Both doctors are experts in their own procedure, with some understanding of the other procedure, but no formal cross-training or expertise in the other procedure. All patients with this disease are diagnosed by Doctor A and discuss treatment with Doctor A first, only having the option of seeing Doctor B and learning about procedure B after receiving a referral from Doctor A. Both procedures were described as having similar costs, and seeing Doctor B would not delay treatment. The words cancer, surgery, radiation, or chemotherapy were not mentioned in the scenario to avoid priming. Respondents were then shown four different treatment discussion approaches (Table 1) by Doctor A and asked to rate the acceptability of each approach on a Likert-type scale ranging from one (completely unacceptable) to five (completely acceptable).

**Table 1:** Summary of Treatment Discussion Approaches Presented to Participants

Approach #	Description
Approach 1	Doctor A discusses only Procedure A. No mention of Procedure B is made.
Approach 2	Doctor A discusses both Procedure A and Procedure B, but expresses preference for Procedure A. No referral is offered.
Approach 3	Doctor A discusses both Procedure A and Procedure B, favors Procedure A, but offers a referral for the patient to see Doctor B for further discussion of Procedure B.
Approach 4	Doctor A routinely refers all patients to Doctor B. Each doctor discusses only their own procedure (Doctor A discusses Procedure A; Doctor B discusses Procedure B).

The second section of the survey sought to assess how differences in efficacy and severe side effects would impact respondents’ views (as patients) of the importance of having alternative treatment options discussed with them. Respondents were presented with hypothetical statistics about the cure rate and severe side effects of both Procedure A and Procedure B. The three variations in cure rate included: Procedure A 80% vs. Procedure B 75%; Procedure A 80% vs. Procedure B 50%; and Procedure A 20% vs. Procedure B 15%. The three variations in severe side effects risks included: Procedure A 5% vs. Procedure B 5%; Procedure A 9% vs. Procedure B 3%; and risk of death from Procedure A 2% vs. Procedure B 0%, although the overall risk of severe side effects is the same. With these variations, a total of nine different scenarios (3x3) were assessed. Respondents were asked to rate the importance of Procedure B being presented as an alternative to Procedure A in each of these scenarios on a Likert-type scale ranging from one (not at all important) to five (extremely important). The final section of the survey collected demographic information from the respondents. A preliminary version of the survey was reviewed for readability and clarity by a cohort of healthcare professionals similar to the target study population prior to its use.

Survey distribution

In July 2022, recruitment emails with the survey link were sent by the principal investigator to all healthcare workers in the participating cancer center’s online directory. This list included physicians (including faculty, residents and fellows), researchers, graduate students, nurses, therapists, administrators, social workers, case managers, and pharmacists. Medical student members of the oncology interest groups at Robert Wood Johnson School of Medicine and New Jersey Medical School were also invited to participate. Of note, our goal in surveying these populations of individuals was to obtain a variety of perspectives from individuals across the care spectrum, ranging from those who are directly involved in therapeutic decision-making (e.g. faculty and trainee physicians), to allied health professionals who care for patients but do not lead informed consent discussions (e.g. nurses, therapists, social workers), to those who do not directly care for patients but promote health outcomes (e.g. researchers). Although patients were not directly invited to participate, some of the surveyed individuals are in many ways closer to patients than "providers." While an individual’s responses to the survey questions is likely to be guided to some extent by their health-related occupation and differ somewhat from the views of a patient [20-22], it was thought that their familiarity with the healthcare system may facilitate more thoughtful responses than those of the general public. Furthermore, it is expected that all of the surveyed healthcare workers have also sought healthcare for themselves or a close family member in the past and would be capable of envisioning themselves in the role of a patient for this study, independent of their profession. In total, 1,683 unique individuals were invited to participate. Non-responders were sent up to three reminder emails. Responses were anonymous and participation was voluntary, without any incentive offered for participation. The study was approved by the Institutional Review Board.

Statistical analysis

Descriptive statistics were used to characterize answers to the Likert-type questions in the survey. The Wilcoxon signed-rank test was used to compare differences between responses to the four treatment discussion approaches. Cohen’s d was used to calculate the standardized effect size between the mean Likert scores of Approaches #2, 3, and 4 in reference to Approach #1. The Friedman test was used to compare responses to variations in cure rate and severe side effect rate in groups of three, holding cure rate constant while evaluating the impact of variations in side effects, and then holding side effect rate constant while evaluating the impact of variations in cure rate. The Mann-U-Whitney and Kruskal Wallis H tests were used, as appropriate, to evaluate differences in responses based on respondents’ gender (men vs. women), race/ethnicity (White vs. Asian vs. underrepresented in medicine (URM)), student status (student vs. non-student), occupation (physician vs other healthcare workers), level of medical training (attending vs. resident/fellow vs. medical student), and subspecialty (surgical vs. medical vs. radiation oncology). Multivariate analyses were not conducted due to the small sample sizes of each subgroup. Findings with p-values less than 0.05 were considered statistically significant. The study was reviewed and approved as exempt from human subjects review by the cancer center’s Institutional Review Board.

## Results

Of 1,683 healthcare workers, 266 responded to the survey (16% response rate). Table 2 shows the demographics of the survey respondents. A total of 166 (62.4%) respondents were women, 161 (56.5%) were white, and 66 (24.8%) were physicians (including residents and fellows) from 17 medical specialties (25 medical oncology, 22 surgical oncology, 8 radiation oncology, 11 other).

**Table 2:** Participant demographics (N=266)

Characteristics	n (%)
Gender	
Men	95 (35.7%)
Women	166 (62.4%)
Not reported	5 (1.9%)
Race	
White or Caucasian	161 (56.5%)
Black or African American	22 (7.7%)
American Indian or Alaska Native	0 (0%)
Asian	57 (20%)
Native Hawaiian or Other Pacific Islander	0 (0%)
Middle Eastern or North African	10 (3.5%)
Other or not reported	16 (5.6%)
Hispanic, Latinx, or Spanish ethnicity	
No	230 (86.4%)
Yes	31 (11.7%)
Not reported	5 (1.9%)
Highest level of education completed	
High school or equivalent	2 (0.8%)
Technical or occupational certificate	5 (1.9%)
Associate degree	10 (3.8%)
Some college coursework completed	9 (3.3%)
Bachelor's degree	64 (24.1%)
Master's degree	44 (16.5%)
Doctorate degree (e.g. PhD)	61 (22.9%)
Professional degree (e.g. MD)	66 (24.8%)
Not reported	5 (1.9%)
Currently a student	
No	220 (82.8%)
Medical student	28 (10.5%)
Undergraduate student	2 (0.8%)
Graduate student	11 (4.1%)
Other type of student or not reported	5 (1.9%)
Role	
Researcher (e.g. basic science or public health)	64 (24.1%)
Attending physician	45 (16.9%)
Administrator	37 (13.9%)
Nurse or advanced care provider	26 (9.8%)
Resident or fellow	21 (7.9%)
Therapist	5 (1.9%)
Social worker or case manager	4 (1.5%)
Pharmacist	1 (0.4%)
Other or not reported	63 23.7%)
Ever held any leadership position in department/division	
Yes	73 (27.4%)
No	153 (57.6%)
Not applicable	35 (13.2%)
Not reported	5 (1.9%)
Have you as a patient ever been faced with a difficult treatment decision?	
Yes	93 (35.0%)
No	171 (64.2%)
Not reported	2 (0.8%)

**Figure 1:**
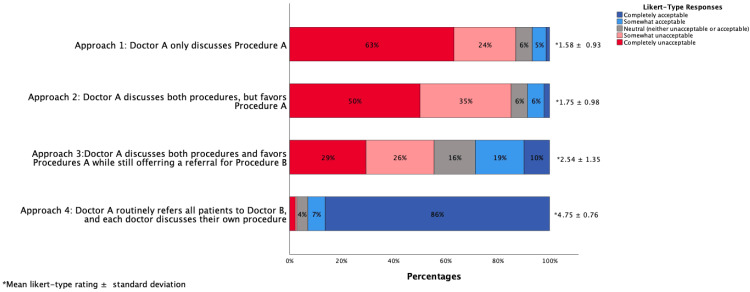
Respondents’ ratings of the acceptability of each of the approaches on a five-point Likert scale.

Figure 1 shows that respondents found Approach 4 (Doctor A routinely refers all patients to Doctor B to discuss Procedure B) to be the most acceptable (mean Likert-type rating 4.75±0.76 (standard deviation)) followed by Approach 3 (2.54±1.35), Approach 2 (1.75±0.98), and Approach 1 (1.58±0.93). The differences between all the approaches were statistically significant (p<0.01). The effect size (Cohen’s d) with Approach 1 as reference was 0.18 for Approach 2, 0.83 for Approach 3, and 3.74 for Approach 4.

On subgroup analysis (Table 3), the rankings across all approaches were identical (4>3>2>1). However, Asian and URM respondents were significantly more likely than white respondents to give a higher score to Approaches 2 and 3 (p<0.01).

**Table 3:** Subgroup analysis for each of the four approaches. * indicates statistical significance at p<.05. † Values are presented as mean (SD). Group comparisons were performed using the Mann–Whitney U test for two-group comparisons and the Kruskal-Wallis test for comparisons among more than two groups. Test statistics are reported as U or H. URM: Underrepresented in medicine.

	Subgroup	n	Approach 1	Statistic	P-Value	Approach 2	Statistic	P-Value	Approach 3	Statistic	P-Value	Approach 4	Statistic	P-Value
Sex				U=7063	0.140		U=7257	0.273		U= 7468	0.515		U=7665	0.526
	Male	94	1.71 (1.03)	-	-	1.80 (0.98)	-	-	2.59 (1.42)	-	-	4.64 (1.00)	-	-
	Female	166	1.52 (0.89)	-	-	1.68 (0.94)	-	-	2.45 (1.31)	-	-	4.81 (0.58)	-	-
Race/Ethnicity				H=7.02	0.030^*^		H=10.732	0.005^*^		H=13.73	0.001^*^		H=5.57	0.062
	White	141	1.50 (0.92)	-	-	1.54 (0.82)	-	-	2.24 (1.29)	-	-	4.87 (0.50)	-	-
	Asian	54	1.81 (0.99)	-	-	1.98 (1.14)	-	-	2.64 (1.35)	-	-	4.61 (0.96)	-	-
	URM	62	1.6 (0.91)	-	-	1.97 (1.06)	-	-	2.98 (1.37)	-	-	4.59 (1.01)	-	-
Healthcare Profession				H=3.05	0.218		H=2.75	0.253		H=0.94	0.626		H=2.35	0.309
	Physician	66	1.68 (0.90)	-	-	1.77 (0.96)	-	-	2.56 (1.35)	-	-	4.86 (0.58)	-	-
	Researcher	63	1.48 (0.78)	-	-	1.63 (0.97)	-	-	2.38 (1.42)	-	-	4.77 (0.71)	-	-
	All others	120	1.57 (1.02)	-	-	1.78 (0.99)	-	-	2.52 (1.34)	-	-	4.68 (0.89)	-	-
Student				U=4127	0.08		U=3842	0.019^*^		U=4070	0.094		U=4454	0.156
	Yes	44	1.84 (1.16)	-	-	2.02 (1.09)	-	-	2.80 (1.36)	-	-	4.91 (0.36)	-	-
	No	219	1.53 (0.88)	-	-	1.68 (0.94)	-	-	2.43 (1.35)	-	-	4.72 (0.81)	-	-
Stage of Training				H=2.61	0.272		H=1.91	0.384		H=2.94	0.230		H=0.75	0.688
	Attending	45	1.56 (0.81)	-	-	1.68 (0.88)	-	-	2.36 (1.23)	-	-	4.87 (0.63)	-	-
	Resident/Fellow	21	1.95 (1.02)	-	-	1.95 (1.12)	-	-	3.00 (1.52)	-	-	4.86 (0.48)	-	-
	Medical student	28	1.93 (1.39)	-	-	1.93 (0.94)	-	-	2.61 (1.23)	-	-	4.96 (0.19)	-	-
Field of Oncology				H=0.50	0.779		H=2.87	0.239		H=1.38	0.501		H=4.81	0.09
	Radiation	8	1.50 (0.53)	-	-	1.63 (1.19)	-	-	2.00 (1.07)	-	-	4.63 (0.74)	-	-
	Medical	25	1.84 (1.03)	-	-	1.96 (1.02)	-	-	2.68 (1.38)	-	-	4.76 (0.83)	-	-
	Surgical	22	1.64 (0.85)	-	-	1.62 (0.97)	-	-	2.55 (1.47)	-	-	5.00 (0.00)	-	-

Table 4 shows respondents’ views on how important it would be for themselves as patients to have Procedure B discussed as an alternative to Procedure A for various permutations in the potential for cure and severe toxicity of each procedure. In eight of the nine scenarios, most respondents felt that it would be very or extremely important to them as the patient that Procedure B be discussed as an alternative to Procedure A. The scenario in which there was a small risk of death from Procedure A, even when severe side effects are otherwise comparable to Procedure B, was associated with significantly more respondents believing that Procedure B should be discussed as an alternative, regardless of the cure rate categories (p<0.01). There was no significant difference in ratings for any toxicity category when comparing procedures with a relatively high cure rate (80% vs. 75%) vs. relatively low cure rate (20% vs. 15%).

**Table 4:** Mean Likert-type score, and percentage of respondents who gave a Likert-type score of 4 or 5 (“very important” or “extremely important”) when asked how important it was for Procedure B to be discussed as an alternative to Procedure A with various combinations of probability of cure or severe toxicity. † P-values were calculated using the Friedman test to compare repeated Likert-scale responses across treatment scenarios.

		Cure Rate				
		80% (A) vs. 75% (B)	80% (A) vs. 50% (B)	20% (A) vs. 15% (B)	Statistic	p-value
Severe Toxicity Rate	5% (A) vs. 5% (B)	3.58 (58.4%)	2.85 (34.6%)	3.52 (57.2%)	X^2^=141.1	<0.001
	9% (A) vs. 3% (B)	4.29 (86.1%)	3.64 (62.3%)	4.13 (79.0%)	X^2^=110.6	<0.001
Death Rate	2% (A) vs. 0% (B)	4.46 (90.6%)	3.91 (70.2%)	4.30 (84.7%)	X^2^=89.2	<0.001

On subgroup analysis, relatively few significant differences were observed. However, when there was a large difference in cure rate (80% vs. 50%), physicians were significantly less likely than all other healthcare workers to believe that Procedure B should be discussed as an alternative treatment option when the severe toxicity rate was the same (mean Likert-type rating 2.55 vs. 2.99, respectively, p=0.02), higher for Procedure A (3.40 vs. 3.77, respectively, p=0.02), or when there was a risk of death for Procedure A (3.74 vs. 3.98, respectively, p=0.05). 

## Discussion

An unbiased discussion of the risks, benefits, and reasonable alternatives to a given treatment is essential to an informed consent process that values patient-centered decision-making. Explaining the risks and benefits of surgery, radiation, and systemic therapy is typically well within the purview of a team of surgical, radiation, and medical oncologists. However, it is unrealistic, given the separation of training and certification in these practice disciplines, to rely on a single individual from any one of these disciplines to have a sufficiently thorough understanding of alternative treatment modalities that fall outside of their scope of training and practice, to provide the information necessary for truly informed patient decision-making. Despite this, it is common practice for oncologists not to provide patients with opportunities to learn about alternative treatment modalities from the physicians with expertise in them [11-18]. Our study suggests that the majority of providers and other types of healthcare professionals would not consider this practice acceptable if they themselves were the patients. Rather, they would strongly favor a more balanced approach in which patients have a full opportunity to discuss their treatment options with the specific member of the team with expertise in each option. Furthermore, our findings suggest that a comprehensive multidisciplinary discussion of treatment options is still preferred even in those situations in which one option may have somewhat inferior efficacy to another, depending on each treatment’s potential for severe side effects.

The hypothetical scenarios that respondents were asked about in this study were intended to reflect those of the real world and these examples are ubiquitous. For instance, high-level evidence supports either surgical resection or radiation therapy as equally effective primary curative treatment modalities for many appropriately selected patients with prostate, bladder, pharyngeal, laryngeal, and lung cancers [23]. Management of certain lymphomas may involve decisions between incorporating radiation with chemotherapy or using more intensive chemotherapy alone. Palliation of symptoms from advanced/metastatic disease may also involve complex decision-making between surgery, radiation, and other invasive procedures like stenting, ablation, or embolization. In some cases, randomized evidence exists to support a given treatment approach for the “average” patient, whereas in other cases there is more of a grey zone in which a physician’s subjective interpretation of observational data and their personal experiences may guide management. In either situation, our data suggests that a discussion of only the “most effective” approach is unacceptable when other options have comparable efficacy, and is especially problematic if that more effective approach also carries a higher risk of severe toxicity or death, even if that risk is relatively small. Of note, although the scenarios in this study were developed with oncology in mind, they are equally applicable to cardiovascular disease or other medical conditions that can be managed in different ways by different subspecialists.

The issues raised herein fall within the conceptual framework of shared decision-making, in which clinician(s), patients and their families jointly participate in a health decision and consider not only the benefits and risks of available treatment options, but also a patient’s values, preferences, and circumstances [24]. Both evidence-based medicine and patient-centered communication skills contribute to shared decision-making. However, incorporating patient values into decision-making is the more challenging, and less studied, component [25]. There are inherent power and information asymmetries in the patient-clinician relationship [26]; without shared decision-making, clinicians are more likely to erroneously guess patient preferences based upon their own biases, and authentic evidence-based medicine will not occur [24,27]. The potential for a clinician’s views to impact practice is also evident in our subgroup analyses, where occupation and race/ethnicity impacted responses to some extent. There is a large body of literature assessing how patients value efficacy in relation to toxicity of cancer treatment [28]; while most patients tend to value quality-of-life more than overall survival or progression-free survival, these values are not universal and can vary considerably based on the treatment intent (curative vs. palliative) and expected outcomes from treatment [29-33]. Although some patients may not want to participate in their healthcare decisions, all patients deserve the opportunity to participate actively in a shared decision-making approach free of others' assumptions and biases that may impact the treatment options that physicians lead a patient to consider [34,35]. 

Strategies to overcome the barriers to multidisciplinary patient-centered care may take a variety of forms. First, the techniques to accomplish shared decision-making should be a component of undergraduate and graduate medical education [36]. Along these lines, further efforts should be made to facilitate cross-disciplinary education among medical, surgical, and radiation oncologists in the United States, which is typically more compartmentalized than in international training programs [8]. The addition of information on how to communicate evidence or incorporate patients’ preferences would also be a valuable addition to mainstream clinical practice guidelines [37,38]. Health systems should also make efforts to promote patient-centered care, for instance through establishment of multidisciplinary clinics that efficiently facilitate access to multiple types of oncologists in a single setting [39-41] Furthermore, developing physician compensation models that reward team-based goals (rather than conventional individual or department-based goals related to number of patients treated or revenue generated) also has the potential to facilitate a more patient-centered culture [42,43].

There are several important limitations to this study. In any survey of this nature, there is the possibility that demand characteristics of the questions may lead participants to respond based on what they think the research is about, or in the socially desirable way. The response rate was low, suggesting potential selection bias in the responses we received. Furthermore, this was a single-center study, and therefore the findings may reflect a collective culture that is reinforced by hospital trainings, didactics, and residency programs in one setting. It also cannot be known for certain whether the findings in this study may be extrapolated to the general public; although respondents were asked to view themselves as patients in this study, physicians and other healthcare workers may have been biased by their occupational roles in healthcare [20-22]. That said, the diverse spectrum of health-related professions and education levels encompassed in our population of respondents allowed for some ability to capture biases or variable attitudes that may have existed, and ultimately we feel that it is likely that the opinions expressed are relatively representative of those employed outside of a healthcare setting as well. However, the survey would need to be administered to patients to evaluate this, since they are the ultimate decision-makers. It should be noted that the demographics of respondents is similar to U.S. healthcare occupations as a whole, in terms of sex, race, and ethnicity [44]. Finally, there are also subtleties to the hypothetical scenarios used that could not fully be explored; for instance, the logistics of obtaining each treatment modality, or the type of toxicity of each treatment modality may have had as much of an impact on individual decisions as the absolute rate of severe toxicity.

## Conclusions

Most healthcare professionals value a balanced, multidisciplinary approach to treatment discussions, even when one option may appear more effective or carries different risks. Respondents overwhelmingly favored scenarios in which patients could hear directly from experts in each relevant specialty, reinforcing the importance of collaboration and transparency in patient care. This preference persisted across professional backgrounds and demographic subgroups, suggesting a broadly shared belief in the ethical and practical value of shared decision-making. Emphasizing multidisciplinary education, cross-specialty communication, and team-based models of care may be essential steps towards ensuring that treatment decisions truly reflect patient-centered values.

## Appendices

**Table 5:** Questionnaire Credits: Marco A. Santos Teles, George Gayed, Vadim M. Shteyler, Richard J. Contrada, Dirk Moore, Francis Barchi, Paul R. Duberstein, Malcolm D. Mattes

Question	Scenario	Response Options
How acceptable to you is Doctor A's approach in each scenario?	Approach 1: Doctor A only discusses Procedure A with you, and you decide to undergo Procedure A without ever seeing Doctor B or discussing Procedure B.	1 – Completely unacceptable, 2 – Somewhat unacceptable, 3 – Neutral (neither unacceptable nor acceptable), 4 – Somewhat acceptable, 5 – Completely acceptable
	Approach 2: Doctor A discusses both Procedures A and B but shares their opinion that Procedure A is more likely to cure your illness than Procedure B (even though objective data suggest they are similar). You decide to undergo Procedure A without seeing Doctor B.	
	Approach 3: As in scenario #2, Doctor A discusses both procedures A and B with you, but shares their opinion that procedure A is more likely to cure your illness than procedure B (even though objective data suggests they are similar). Doctor A also suggests a referral to Doctor B to discuss procedure B, but based on the information given by Doctor A, you decide not to see Doctor B, and undergo procedure A.	
	Approach 4: Doctor A routinely refers all patients with your illness to Doctor B. You have consultations with both doctors and discuss both procedures with each. For this study, you decide to undergo Procedure A.	
For each scenario, how important is it to you that Procedure B be presented to you as a reasonable alternative to Procedure A?	If the cure rate is slightly higher for Procedure A (80% vs. 75%) and the risk of severe side effects is the same (5%) for both procedures.	1 – Not at all important, 2 – Slightly important, 3 – Moderately important, 4 – Very important, 5 – Extremely important
	If the cure rate is slightly higher for Procedure A (80% vs. 75%) and the risk of severe side effects is higher for Procedure A (9% vs. 3%).	
	If the cure rate is slightly higher for Procedure A (80% vs. 75%) and the risk of death during the procedure is higher for Procedure A (2% vs. 0%).	
	If the cure rate is much higher for Procedure A (80% vs. 50%) and the risk of severe side effects is the same (5%) for both procedures.	
	If the cure rate is much higher for Procedure A (80% vs. 50%) and the risk of severe side effects is higher for Procedure A (9% vs. 3%).	
	If the cure rate is much higher for Procedure A (80% vs. 50%) and the risk of death during the procedure is higher for Procedure A (2% vs. 0%).	
	If the cure rate is much lower for both procedures, but slightly higher for Procedure A (20% vs. 15%) and the risk of severe side effects is the same (5%) for both.	
	If the cure rate is much lower for both procedures, but slightly higher for Procedure A (20% vs. 15%) and the risk of severe side effects is higher for Procedure A (9% vs. 3%).	
	If the cure rate is much lower for both procedures, but slightly higher for Procedure A (20% vs. 15%) and the risk of death during the procedure is higher for Procedure A (2% vs. 0%).	
